# Deep learning-assisted structural color inverse design: a perspective

**DOI:** 10.1039/d6sc01773h

**Published:** 2026-07-01

**Authors:** Taigao Ma, L. Jay Guo

**Affiliations:** a Department of Physics, The University of Michigan Ann Arbor MI 48109 USA; b Department of Applied Physics, The University of Michigan Ann Arbor MI 48109 USA guo@umich.edu; c Department of Electrical Engineering and Computer Science, The University of Michigan Ann Arbor MI 48109 USA

## Abstract

Structural color, a special color generated by the interaction of visible light with nanostructures, has attracted significant research interest and industrial attention due to its promising advantages of long-term stability, spatial resolution, sustainability, and multi-functionality. The rapid development of artificial structural coloration has also facilitated extensive applications, including colored filters, coatings, high-resolution color printing, information encryption, sensing, *etc.* A critical challenge in these applications is how to design nanostructures with the desired color properties. Recently, deep learning has emerged as a powerful tool for the inverse design of structural color, demonstrating superior performance over traditional optimization-based methods. However, current approaches have not yet fully exploited the capabilities of deep learning. In this perspective, we hope to fill this gap by giving a summary of current research frontiers and providing insights into how deep learning can be further leveraged to advance the structural color design, from basic representations of colors and structures, to novel structural colors empowered by deep learning, and also pointing out new opportunities that deep learning can potentially bring to this research domain. We hope this perspective can inspire new ideas and further development for structural color design in the future.

## Abstract 2

This paper summarizes how to combine deep learning in structural color inverse design, from basic representations of colors and structures, to novel structural colors applications as well as new opportunities.

## Graphical abstract

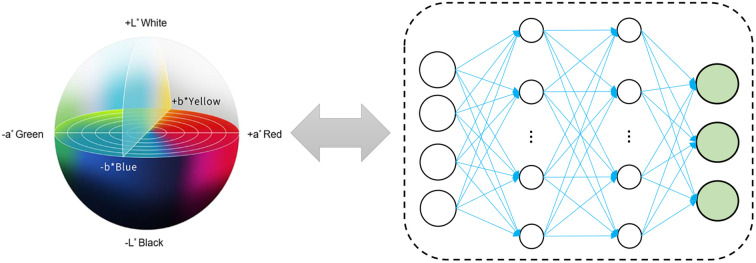

## Introduction

Color is ubiquitous in our daily lives. It plays a fundamental role in human perception and enriches our interaction with the surrounding world. Traditionally, color generation relies on light-absorbing materials such as organic dyes and chemical pigments. However, these materials are usually not stable and can degrade gradually when exposed to heat or sunlight. In addition, they suffer from the issue of low spatial resolution, limited color gamut and brightness, and potential environmental concerns. These challenges have motivated researchers and engineers to search for alternative solutions for color generation: structural color.

Different from dyes or pigments, structural color comes from the interaction of light with nanoscale structures,^1–8^*e.g.*, interference, diffraction, or scattering. These phenomena can be observed in nature, such as in peacock feathers^9^ and beetles,^10^ or can be artificially generated by engineering nanostructures, *e.g.*, multilayer structures,^11,12^ photonic crystals,^13,14^ metasurfaces,^2,15,16^ plasmonics,^17–20^ and particles,^6,21–23^ to manipulate light–matter interaction. Structural colors offer superior advantages, including high spatial resolution, enhanced brightness, long-term stability, dynamic-switching ability, and environmental friendliness. Because of this, they have shown great potential in multiple industrial applications, such as color printing, displays, information encryption, decorations, optical sensing.

Traditionally, the design of nanostructures for targeted structural colors has heavily relied on iterative optimization-based methods.^24–26^ These methods involve simulating the optical performance of candidate structures at each optimization step and then iteratively refining the design based on the evaluation metrics, *e.g.*, minimizing the color difference between candidate structures and the design target. However, this process will be computationally inefficient when searching inside a large design space. Furthermore, most optimizations are heuristic during the searching process and do not learn from the design process, so they may fail to identify high-performance designs. These challenges have motivated the exploration of data-driven deep learning models to accelerate and automate the inverse design process for structural color generation. Different from optimization, deep learning methods use neural networks to learn the inverse mapping from color space to the corresponding structure space. Once trained, these models can generate structures with high performance within 1 millisecond, significantly accelerating the design process. Up to now, various types of neural network architectures have been developed and successfully applied to solve this task, including tandem networks, Generative Adversarial Networks (GANs), Mixture Density Networks (MDNs), Recurrent Neural Networks (RNNs), transformers, *etc.*

Considering the rapid advancements in this field, it is essential to summarize current research progress and provide insightful instructions for both newcomers and experienced researchers. Although there are already many good reviews published for deep learning-based inverse design on photonic and nanophotonic systems,^12,27–35^ to the best of our knowledge, none of them have specifically focused on deep learning-based structural color inverse design. We hope to use this perspective to fill in this gap. In addition, we also noticed that existing reviews in related fields have already covered many fundamental topics, including different types of neural network models and their architectures, dataset creation, model construction, and the training and evaluation pipeline. There are also many nice benchmark papers that systematically compared the performance of these neural network models in a fair setting.^36–39^ Rather than revisiting these well-covered aspects (while we do encourage interested readers to read and explore prior studies first), in this perspective, we will instead focus more on the unique interplay between deep learning and structural color. Our discussions will identify emerging new opportunities and highlight novel applications to advance this research domain.

This perspective is organized as follows. In the first part, we will examine different methods to represent colors as the design target for neural network input. Although color space representation is the most straightforward method, recent advances in color-to-spectrum conversion look like a promising alternative because it allows us to design colors based on a spectrum. We would like to highlight the potential of this new spectrum representation as it shows great possibility for downstream color-based applications. In the second part, we will provide a systematic overview on how to represent nanostructures as geometrical parameters for neural network outputs. We categorize existing methods into three distinct paradigms: vectorized representation, pixelated representation, and serialized representation. Each of them corresponds to some specific types of neural network architectures. In the third part, we will explore novel structural color applications empowered by deep learning that extend beyond conventional color filters. By showcasing these emerging use cases, we aim to inspire future researchers to leverage the superior performance of deep learning and bring new capabilities to structural color. Finally, in addition to the faster design speed and improved color accuracy, we have investigated and identified some additional opportunities that deep learning can bring to enhance color design, including design diversity, structural robustness, and design explainability. We hope this perspective can provide readers with fresh insights and stimulate new ideas and thinking in structural color design in the future.

## Input: color representation

**Fig. 1:**
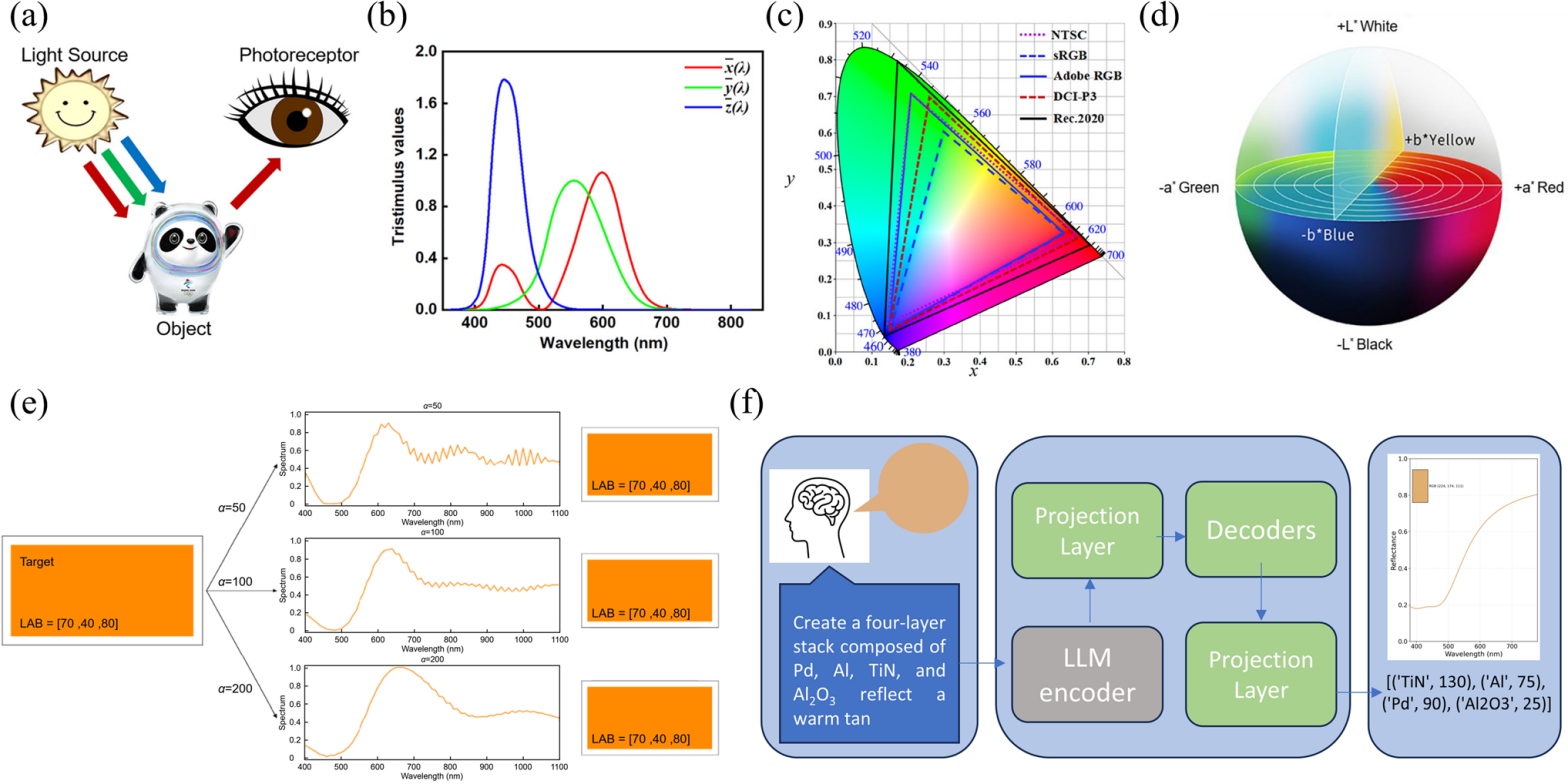
Different types of color representations. (a) Schematic diagram of the color perception process, where objected-reflected light from the light source is captured by the human retina and generates visual signals for color perception. (b) Three color matching functions used to convert the spectrum to color coordinates, including *x̄*(*λ*), *ȳ*(*λ*), *z̄*(*λ*). (c) The CIE 1931 XYZ chromaticity diagram and five popular color gamut standards. (d) The CIE 1976 L^∗^a^∗^b^∗^ chromaticity diagram with a uniform color space. (e) Illustration of converting L*a*b* color to any spectrum region using a different *α* factor. As we increase *α*, the converted spectrum will be smoother. (f) Schematic diagram of using natural language description for structural color representation, which provides a conversational interface for color inverse design. (a–d) Reprinted from Wang *et al.*^2^ Copyright @2023, De Gruyter. (e) Reprinted from Ma *et al.*^48^ Copyright @ OE Journals Group. (f) Reprinted from Li *et al.*^50^ Copyright @ The Thirty-Ninth Annual Conference on Neural Information Processing Systems.

Before discussing any specific methodologies, it is important to figure out how to obtain suitable representations for structural colors because they are critical inputs to neural networks. As shown in Fig. 1(a), color itself is a subject perception of human eyes under the surrounding light source,^40,41^ including hue, saturation, and brightness.^42–44^ Conventionally, this perception can be quantified by using three-dimensional color spaces, including RGB, XYZ, xyY, and L*a*b* color space,^2,45–47^ and dominates the current literature on color design. However, color spaces are highly compressed values for color perception because they are obtained by converting from the reflection/transmission spectrum in the visible region (400 nm to 800 nm). Therefore, they may not provide sufficient information to guide the design process, *e.g.*, aiming towards multi-functional structural color. A more fundamental approach for color design is to use spectral representation directly.^48,49^ However, we found that this method has not yet been extensively explored because it requires converting the three-dimensional color coordinates back to the high-dimensional spectrum. In addition, benefiting from the recent fast development of large language models, human-readable natural language descriptions for structural color have also been proposed to provide a flexible and user-friendly conversational interface for inverse design.^50^ We would like to draw the reader's attention to these color representations for future research.

### Color space representation

There are two color spaces that are widely used in structural color inverse design: the CIE 1931 XYZ color space and the CIE 1976 L*a*b* color space.^47,51^ These three value coordinates in CIE 1931 XYZ color space can be obtained by integrating the perceived spectrum of the target object with three color matching functions (to represent the eye's response to the R, G, B, colors; see Fig. 1(b)) and spectral power distribution of illuminating light. After performing a normalization on the first two values, their associated coordinates (*x*, *y*) represent the color gamut and can be visualized in a 2D perceptive color map as shown in Fig. 1(c). However, designing in XYZ color space may lead to difficulties when comparing the color difference because this color space is not uniform. Therefore, many researchers use CIE 1976 L*a*b* color space to obtain a more uniform color difference. L*a*b* color space can be converted from XYZ color space by doing a simple mathematical calculation.^2^ As shown in Fig. 1(d), L^∗^ represents the brightness level of this specific color, while a^∗^ and b^∗^ incorporate the color's chromaticity information. Δ*E* can be used to evaluate the color difference that matches human eyes. Generally, two colors with Δ*E* ≤ 2 can be treated as the same color because such a small visual difference cannot be distinguished by human eyes.

### Spectrum representation

Instead of using color space for color representation, the spectrum itself can also be used for color representation. For example, one can use the discretized visible spectrum from 400 nm to 800 nm as the design target. Usage of either the reflective-type or transmissive-type spectrum depends on the specific design needs. There are multiple advantages of doing so. First, it is more physical and straightforward to design colors based on a spectrum. This is because a spectrum is a natural characteristic of nanostructures after solving physical equations, while extra subjective conversions and calculations are required to obtain color coordinates. Second, a spectrum provides more physical information to guide the design process and helps to explain why the designed nanostructure exhibits the desired color performance, *e.g.*, resonance behaviors and absorption peak. Finally, a spectrum in the visible region can be directly extended to other wavelength regions for multi-functional structural color applications. For example, simultaneous design of visible and near-infrared spectra enables potential applications for colored solar panels^52^ and colorful radiative cooling devices.^53^ It will be more flexible for researchers to tailor the target spectrum in multiple spectral bands to achieve their specific needs.

Despite these advantages, spectrum representation has not been widely explored in structural color inverse design. The major challenge comes from the difficulty of obtaining the corresponding spectrum for a specific color, which usually requires solving the problem of inverse mapping from low-dimensional color space to high dimensional spectrum space. Recently, Ma *et al.*^48^ proposed an optimization-based method to convert colors to spectra within the desired wavelength region, opening up a new possibility for this direction. For a given color target LAB_target_ in the LAB space, they want to find a spectrum S where its converted color coordinate LAB_*S*_ is close to LAB_target_ while making sure the spectrum is physical. In their methods, they treat the inverse conversion as a combined optimization task with two goals. The first goal is to minimize the difference between the color of the converted spectra and the target color, *i.e.*, Δ*E*(LAB_target_, LAB_*S*_). The second goal is to minimize the second order derivatives of the converted spectra, which is denoted as 
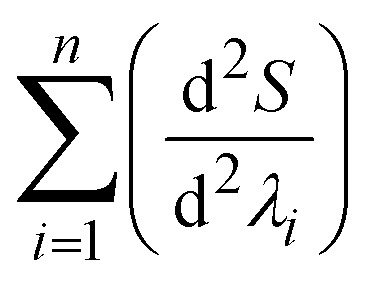
, and *λ*_*i*_ is the *i*_th_ wavelength point in the discretized spectrum. This helps to remove abrupt jumps in the adjacent spectrum and ensure that the converted spectra are physical. Therefore, they seek to minimize the following loss:
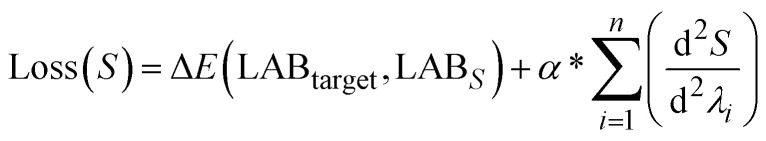


In this loss function, the first term corresponds to the color difference and the second term quantifies the spectrum smoothness, where *α* is the factor balancing these two loss functions. Notice that the color difference in the first term will only be calculated within the visible region (in this case, from 400 nm to 700 nm), while the second term is calculated in the extended spectral region (in this case, from 400 nm to 1100 nm). This method provides extra flexibility to convert color to spectra in a larger wavelength region for other applications. As an example, Fig. 1(e) shows an illustration of converting an orange color into continuous spectra with different *α* coefficients. We can tell that as *α* is increasing, the converted spectra will become smoother. The high broadband peak at 650 nm also explains why the color is orange. Using this method, the authors have successfully designed multiple structural colors in both reflective and transmissive types, and use a single neural network model to unify the design of structural color and general spectrum design.

### Natural language representation

Recently, with the rapid development of large language models (LLMs), natural language has emerged as a promising interface to bridge the gap between humans and science, including chemistry,^54–56^ biomedical research,^57–59^ materials,^60,61^ algorithm design,^62^ nanophotonic design,^63^ automation,^64^*etc.* While color space and spectra are highly precise and quantitative, compared to natural language, they lack the intuitive flexibility for some complicated and multi-objective design scenarios such as specifying material preference, layer constraints, or angular performance.

Recently, Li *et al.*^50^ proposed a framework called Conversational Human-Readable Optical Multilayer Assembly (CHROMA), which represents the color target as a natural language description and treats the inverse design process as a translation task from human-readable text to a discrete sequence of materials and thicknesses. As shown in Fig. 1(f), when a user wants to design a reflective tan color using specific materials, he can directly input a detailed description about the color target to the model: “Create a four-layer stack composed of Pd, Al, TiN, and Al_2_O_3_, reflect a warm tan”, and obtains the designed multilayer structure directly. This representation method enables the “human-in-the-loop” design with a straightforward descriptive input target, and allows for seamless integration of hard constraints such as materials or structures, directly in the input prompt.

The introduction of natural language representation can significantly lower the barrier to entry for structural color design. It converts the inverse design process from a highly-technical and optimization-intensive task into a simple conversational interaction, providing a flexible, user-friendly toolset that can handle a diverse set of optical requirements and practical fabrication constraints simultaneously. As LLMs continue to evolve, we anticipate that language-driven representations will play a more and more important role in inverse design platforms, enabling researchers and engineers to explore the vast design space of nanostructures through intuitive, multimodal communication.

### Output: nanostructure representation and neural network selection

In the previous section, we discussed that color representation for neural network inputs can be low-dimensional color space, high-dimensional spectral space, or natural language. The former two representations can be formulated as a single vector, while the difference is the dimensionality. Therefore, either using color coordinates or spectral representations, the input to neural networks would always have the same data format. However, things are quite different when we look at the output of neural networks, which corresponds to the structural parameters of nanostructures we intend to design. These parameters are usually of a high degree-of-freedom, including the geometric features of nanostructures (*e.g.*, height, length, width, diameter, and gap), shape and orientation of meta-atoms, number of layers, and material combinations. Furthermore, there are diverse types of nanostructures that can be used for structural color generation, ranging from simple multi-layer thin film structures to periodic gratings and complex metasurface structures and nanoparticle systems.

For a better discussion, we categorize these nanostructure parameters into three fundamental types: (1) vectorized, (2) pixelated, and (3) serialized representation, and each corresponds to some specific neural network architectures. For example, multilayer perceptron (MLP, *e.g.*, tandem networks) is mostly suited for vectorized representation, while Convolutional Neural Networks (CNNs) typically handle pixelated representations. Recurrent Neural Networks (RNNs) and transformers are widely used for sequential representation. In Table 1, we provide a summary on existing deep-learning based structural color inverse design methods, and compare them based on diverse aspects, including color representations, nanostructure systems, selection of neural network architectures and specific applications. We find out that existing research predominately employs vectorized representation and use tandem networks as the inverse design model backbone, meaning the other two types are still underdeveloped. We think this could be a new opportunity and encourage further explorations on these two representations, which may lead to better performance and potentially novel capabilities in structural color design.

**Table 1:** A summary on existing deep-learning based structural color inverse design methods

Ref.	Year	Color representation	Structure representation	Nanostructure system	Neural network architecture	Structural color applications
Gao *et al.*^65^	2019	xyY	Vectorized	Silicon nanorods	Tandem network	—
Huang *et al.*^66^	2019	xyY	Vectorized	Dielectric ring array	MLP	—
Sajedian *et al.*^67^	2019	Lab	Vectorized	Silicon nanodisks	Deep-Q network	—
Dai *et al.*^68^	2021	xyY	Vectorized	FP cavity	cGAN	—
Roberts *et al.*^69^	2021	xyY	Vectorized	Plasmonic metasurfaces	Tandem networks	—
Ma *et al.*^70^	2021	Lab	Vectorized	Nanoparticles	Tandem networks	
Zhou *et al.*^71^	2022	xyY	Vectorized	Nanoparticles	Tandem networks	—
Wang *et al.*^72^	2022	xyY	Vectorized	Silicon nanopillar	LSTM	—
Wang *et al.*^73^	2022	RGB	Vectorized	Multilayer	MDN	—
Hao *et al.*^74^	2022	xyY	Vectorized	Cone silicon	Tandem networks	—
Hu *et al.*^75^	2022	Lab	Vectorized	Hybrid metal–dielectric metasurfaces	Tandem networks	Dynamic
Dai *et al.*^76^	2022	Lab	Vectorized	Multilayer	cGAN	Dynamic
Liu *et al.*^77^	2023	Spectrum	Vectorized	Plasmonic nanohole	Tandem networks	—
Guan *et al.*^78^	2023	Spectrum	Vectorized	Multilayer	Tandem networks	Radiative cooling
Prakash *et al.*^79^	2023	Lab	Vectorized	Metamaterials	Tandem networks	Dynamic
Souza *et al.*^80^	2023	Spectrum	Vectorized	Slanted grating	Multi-valued NN	—
Saha *et al.*^49^	2023	Spectrum	Serialized	Multilayer	RNN	—
Hu *et al.*^81^	2024	Lab	Vectorized	Dielectric metasurfaces	TC-TNN	Dynamic
Yan *et al.*^82^	2024	Lab	Vectorized	Dielectric metasurfaces	Tandem networks	—
Fang *et al.*^83^	2024	xyY	Vectorized	Metasurfaces	Tandem networks	Dynamic
Keawmuang *et al.*^84^	2024	Spectrum	Vectorized	Multilayer	Tandem networks	Radiative cooling
Fang *et al.*^83^	2024	xyY	Vectorized	Metasurfaces	Tandem networks	Dynamic
Zuo *et al.*^85^	2024	RGB	Vectorized	Metasurfaces	Tandem networks	Polarization-independent
Ma *et al.*^48^	2024	Spectrum	Serialized	Multilayer	Transformer	—
Wang *et al.*^86^	2024	Spectrum	Vectorized	Guided mode resonance	Tandem networks	—
Yang *et al.*^87^	2025	RGB	Vectorized	Metasurfaces	Tandem networks	Dynamic
Sadman *et al.*^88^	2025	xyY	Vectorized	Grating	Tandem networks	—
Ma *et al.*^89^	2025	Lab	Vectorized	Multilayer	Tandem networks	Radiative cooling
Li *et al.*^50^	2025	Language	Serialized	Multilayer	Transformer	—

### Vectorized representation

Vectorized representation encodes nanostructure parameters as a multi-dimensional vector. To do this, we need to first pre-define a structure template by fixing materials used at each region and deciding the shape format within the system; then we select the structural parameters that we need to design, including height, thickness, period, gap, width, and diameter, and combine them into one vector. In this case, each dimension of the vector will represent a specific structural parameter, and the total number of dimensions depends on the system of the designed nanostructure and can vary a lot. Since both inputs and outputs are vectors, this inverse mapping relationship can be learned well using MLP-type neural networks, including tandem networks, Generative Adversarial Networks (GANs), and Variational Auto-Encoders (VAEs), and Mixture Density Network (MDN).

**Fig. 2:**
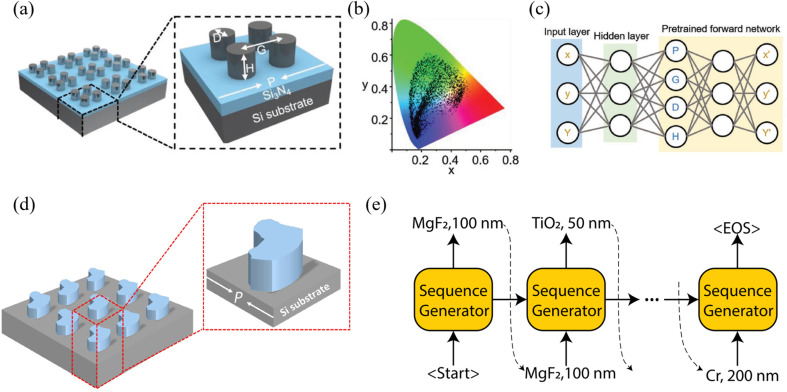
Illustration of different types of structural representations. (a) An example of a vectorized representation for periodic silicon nanorod structures. There are four design parameters: *D* (diameter), *H* (height), *G* (gap), and *P* (period) (b) The color coverage in the CIE chromaticity diagram for the nanostructure shown in (a). (c) Illustration of tandem networks, which includes an inverse neural network for structure design in tandem with a pretrained forward network for optical response simulation. (d) An example of a pixelated representation for a periodic metasurface structure. (e) An example of a serialized representation for a multilayer thin film structure inverse design. (a–c) Reprinted from Gao *et al.*^90^ Copyright @2019, Willey. (d) Reprinted from Ma *et al.*^94^ Copyright @ 2022, Institute of Optics and Electronics, Chinese Academy of Sciences. (e) Reprinted from Wang *et al.*^95^ Copyright @ 2021, IOP Publishing Ltd.

In 2019, Gao *et al.*^90^ proposed a bidirectional neural network called tandem networks, to accurately inverse design silicon structural color. This is the very first work that successfully combines deep learning for color inverse design. As shown in Fig. 2(a), the designed nanostructure is a periodic silicon structure, where each unit cell consists of four silicon nanodisks sitting on top of the silicon substrate with 70 nm Si_3_N_4_ as the index matching layer in between. In this template structure, there are four parameters to design: the diameter (*D*) and height (*H*) of each individual nanodisk, the gap (*G*) between nearby nanodisks in a unit cell, and the period (*P*) of each unit cell. They select xyY as the target color design space and Fig. 2(b) shows the color coverage of the training dataset in the CIE chromaticity diagram. The architecture of the proposed tandem network is given in Fig. 2(c), which has two neural networks connected together: the trainable inverse neural network (INN) and the pretrained forward neural network (FNN). The training of the tandem network includes two steps: (1) initial pretraining of the FNN for accurate structural-to-color prediction and (2) INN training for inverse design by utilizing the frozen FNN to provide feedbacks for color accuracy. Using tandem networks, the authors have demonstrated superior design accuracy and computational efficiency, with design time reduced significantly by orders of magnitude compared to optimization.

Tandem networks are one of the most widely used model backbones for structural color inverse design. Ever since then, many other studies built on a tandem network or its variants have been reported on structural color inverse design, but based on different nanostructures. For example, Dai *et al.*^68^ used a tandem network to design the layer thickness in the Fabry–Perot cavity for structural color filters. This FP cavity structure provides a significantly wider color space that is 215% of sRGB. They chose LAB as the color space for more uniform color representation. Apart from nanostructures, nanoparticles also serve as an important method to generate structural colors based on scattering through localized surface plasmon resonances or Mie resonance. Differently, the structural parameters inside these chemical systems are statistical in nature, such as mean particle size, volume fraction, and spatial arrangement. These parameters can be well described using a vector, where each dimension corresponds to a specific statistic, and therefore tandem networks can also be utilized for their inverse design. For example, Mat *et.al*^91^ utilized a tandem network to design gold nanoparticle structural color. The nanoparticle system contains three design parameters: the particle radius *r*, particle volume fraction *f*_V_, and the layer thickness *h*. They generated a large dataset between nanoparticles and structural color using Mento Carlo and Mie scattering simulations, and then trained a tandem network to accurately predict the nanoparticle design, achieving a high accuracy of 99.83% on the predicted colors. Similarly, Zhou *et.al*^92^ used four parameters to describe silicon nanoparticle systems: the particle effective radius *r*_eff_, particle volume fraction *f*_v_, effective variance *v*_eff_, and layer thickness *h*. A tandem network was also used to design their color properties and achieved good performance. There are also many other studies based on all dielectric metasurface-based substrative color,^72^ colored silicon nanoparticle systems,^71^ truncated cone silicon nanostructures,^74^ silver nanoholes,^77^ hybrid metal and dielectric resonators,^83^ guided mode resonator filters,^86^ gratings,^88^*etc.*

In addition to tandem networks, there are also many other types of neural networks that can be used for vectorized representation-based inverse design. For example, Dai *et al.*^93^ demonstrated that conditional GANs can be used to inverse design for transmissive FP cavity-based structural color devices and can generate sets of multiple solutions to a design problem, instead of only giving one solution as suggested in tandem networks. In ref. 73, Wang *et al.* combined optimization with MDN to obtain a material-aware model for accurate and efficient structural color inverse design based on multilayer thin film structures. To get a better understanding of these models and their relative design performance, we recommend some benchmark papers that compared each model in a fair setting.^36,39^

### Pixelated representation

In pixelated representation, the nanostructure can be denoted as an image pattern on a 2D surface. One example is given in Fig. 2(d). Usually, these nanostructures are periodic metasurfaces with free-form shapes that are surrounded by a medium, *e.g.*, air. For each unit cell, we can represent it as an *M* × *N* pixelated binarized image, where white pixels and black pixels correspond to the surrounding medium and the freeform structure, respectively. Here, *M* and *N* denote the number of pixels along each dimension, and they need to be carefully selected such that the smallest element in this unit cell corresponds to the achievable resolution during fabrication. This type of representation aligns well with convolutional neural networks (CNNs), which takes in the vectorized color information and generates images as the designed structures using convolutional layers. Although CNNs have been widely used for spectrum inverse design, their application to freeform metasurface-based structural color design still remain underexplored, which could be a potential research direction.

### Serialized representation

Beyond vectors and images, some types of nanostructures can also be encoded as sequential data, where each element in the sequence corresponds to a distinct substructure. For example, as shown in Fig. 2(e), we can use a sequence to represent multilayer thin film structures, where each sequence element represents an individual layer with different thicknesses and material properties. This sequential representation offers unique advantages: neural networks can now dynamically decide which sub-structure to add as the next sequence element based on existing designed structures, and also automatically decide when to terminate the design process, therefore enabling more exploration and flexibility during the design process. There are mainly two types of neural networks that are widely used for serialized representation: RNN and transformers.

RNN processes sequential data by maintaining a hidden memory of previous sequential inputs and processing them to obtain the prediction of the next element. As an example, Wang *et al.*^95^ proposed a novel design algorithm called OMLPPO to design an optical spectrum for a multilayer thin film. The neural network architecture for sequence generation is based on one special type of RNN called Gated Recurrent Units^96,97^ (GRU). At each sequential step, the GRU will simultaneously design the thickness and material information for that specific layer. To effectively train this GRU for inverse design, the authors used the reinforcement learning algorithm to maximize the expected cumulative rewards. The reward is defined as one minus the difference between the target optical responses and the simulated optical responses of the currently designed structure. In a later study, Saha *et al.*^49^ utilized this algorithm to design a reflective-type structural color for chrome-look finishing and experimentally demonstrated the design performance.

Transformers^98^ are another type of neural network that can handle the sequential data very well. Different from RNN, transformers process the entire sequence simultaneously, and use the self-attention mechanism to selectively focus on the long-context sequence for better information fusion and extraction, and predict the next serialized item in an auto-regressive way. They serve as the backbone in many large language models for complicated text generation tasks, including ChatGPT,^99^ Deepseek^100^ and LLaMA.^101^ Recently, Ma *et al.*^48^ proposed a transformer-based neural network called OptoGPT, that can take the reflection and transmission spectrum as the input and output the predicted multilayer thin film structure in a serialized representation. In their work, they successfully used OptoGPT for accurate structural color design by first performing a color-to-spectrum inversion and then designing colors in the spectrum space, for both reflection-type and transmission-type structural color. In another study, Li *et al.*^50^ also used transformer-based encoders to process the natural language input and used another transformer-based decoder to output the multilayer structure sequence as the design result.

As a summary, there are multiple types of representations for nanostructures, which fundamentally determines the neural network architecture that we can use for inverse design. Each neural network has their own specialization when processing different types of data formats, from MLP capturing vectorized parameter spaces to RNN and transformers suited for complex sequential relationships. In addition, the recent success of a diffusion model^102^ in image generation also provides extra choices for structural representation. There are already some studies that combine the diffusion model in nanophotonic inverse design,^103–106^ but their application in structural color design is still in the very early stage. We do encourage readers to explore different types of deep learning models and spectrum representations and find the best one that can solve their tasks. If a certain representation does not work well, we always suggest switching to other types of representations.

### Beyond color filters: novel applications

In the previous two sections, we summarized how we should represent the input and output of neural networks as well as the selection of the model architecture based on the output format. In the general framework of using deep learning for structural color inverse design, the next step is to prepare a large dataset for model training. This can be done using commercial simulation tools, including COMSOL, FDTD,^108^ RCWA,^109^ TMM,^110^*etc.* The exact amount of dataset depends on the design difficulty, and ranges from several thousands to several millions. Once the training dataset is constructed, we will then need to conduct extensive experiments on model training and evaluation to find the best neural network model that effectively learns the inverse design process. In this perspective, we do not plan to cover these topics. As long as the input, output, and model architecture are determined for the design task, the rest of the work is standard and can be found in any deep learning book or papers. There are also many open-source code repositories, tools, and tutorials that are well-developed for deep learning model training and evaluation.^111–115^

Instead, we will discuss how we can leverage deep learning for structural color design and achieve novel applications and new capabilities. As shown in Table 1, existing deep learning-based methods mainly explore the structural color application for colored filters. With deep learning, we can consider using it to solve complicated tasks and design new nanostructures with novel applications that are not possible previously. Here, going beyond color filters, we will show multiple examples of how structural colors can be incorporated into other situations to achieve multi-functional photonic devices.

### Colored solar cells

Existing solar photovoltaic (PV) panels are predominately in dark black or deep blue colors in order to guarantee a high energy conversion rate, making them mostly suitable for rooftop installation or large power stations in remote terrains. Integrating PV panels into houses, building, infrastructures, or even automobiles has great potential to bring clean energy to everyday usage and promote a sustainable environment, while still preserving an aesthetically pleasing appearance.^52,116–118^ On one hand, we would like the PV panel to reflect a certain amount of light to so the panel looks colorful in the visible region; on the other hand, to ensure high power conversion efficiency, one needs to minimize the light reflected from the PV panel so that the maximum amount of solar energy can be utilized to generate electrical energy. The development of a colorful solar cell presents a fundamental trade-off of balancing the energy efficiency and color appearance, making the design process very challenging.

**Fig. 3:**
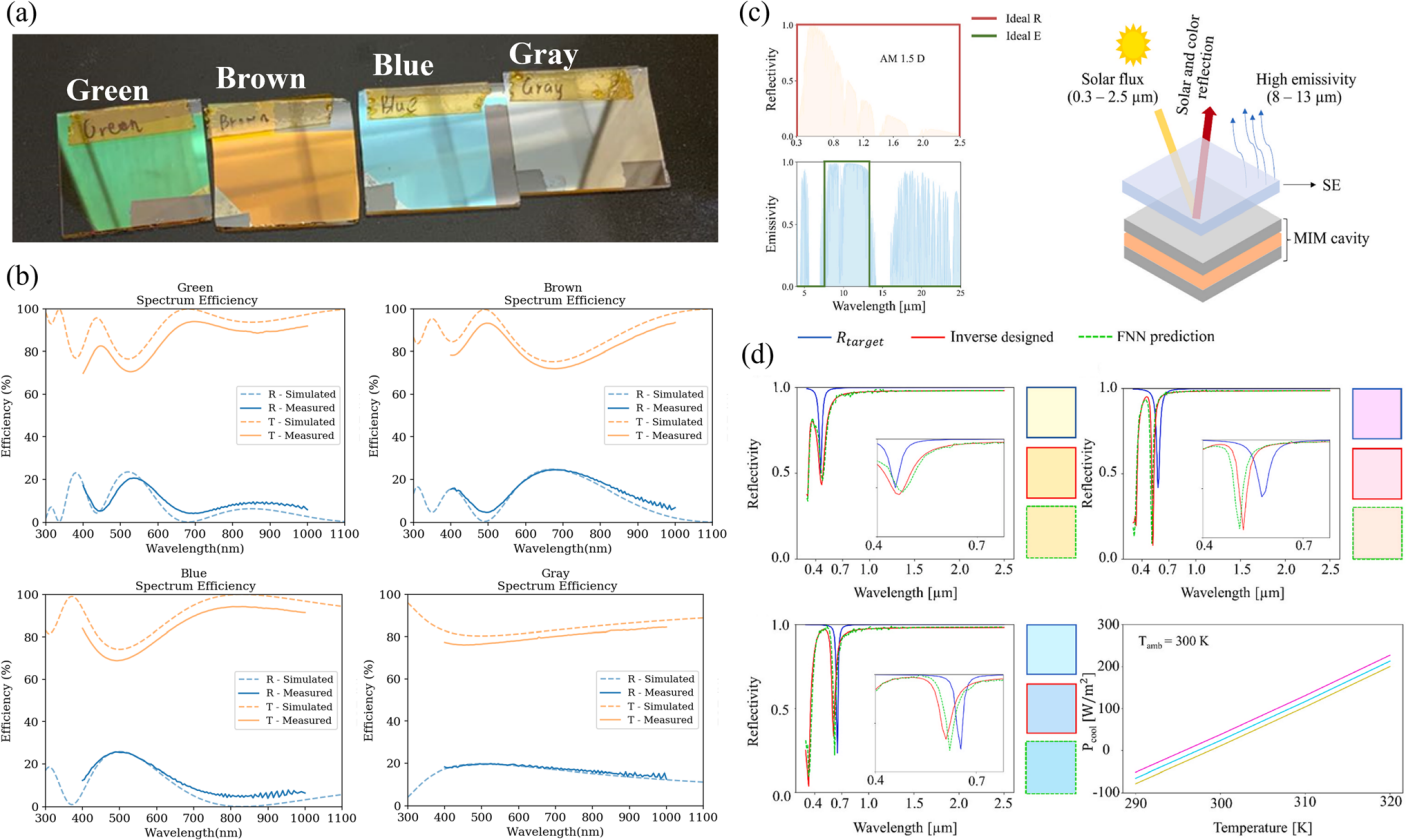
Examples of novel structural color applications. (a) A photo of structural color filters designed for colored solar cells (green, brown, blue, and gray). (b) The simulated and measured transmission and reflection spectra for these color filters shown in (a). All of them exhibit a small reflection peak and high transmission for balanced color appearance and energy efficiency. (c) The ideal optical responses for daytime radiative cooling and their schematic diagram. (d) The reflection spectrum for three different colored radiative cooling and their cooling ability. (a and b) Reprinted from Ma^107^ Copyright @ 2024, University of Michigan. (c and d) Reprinted from Keawmuang *et al.*^84^ Copyright @ 2024, Elsevier.

Recently, in ref. 107, researchers proposed a new method to address this challenge by utilizing reinforcement learning algorithms. Instead of designing a PV panel, they seek to design a colored reflector that can be applied directly on top of any commercial solar cells to make them colorful. Such a colored reflector is based on multilayer thin film structures, and is designed to have a narrowband reflection in the visible region and a high transmission in the solar spectrum, ranging from 400 nm to 1100 nm. Using reinforcement learning, the authors successfully designed four different colors: green, blue, brown, and gray, and experimentally fabricated these colorful reflectors, with sample images shown in Fig. 3(a). Fig. 3(b) gives the details of the measured reflection and transmission spectra. Placing these filters on top of a commercial solar cell turns a black solar cell to a colored one, and the measured photocurrent showed a slight reduction as a function of the applied voltage. Compared with the power conversion efficiency of a reference solar cell having a cover glass on top, these colored solar cells with specially designed filters maintain as high as ≥92% relative energy efficiency, while still retaining an attractive color appearance.

### Colored radiative cooling

Another application of structural color is to provide aesthetic appearance to passive radiative cooling devices. By selectively reflecting the solar irradiance and emitting heat to the universe, these devices serve as a sustainable and cost-effective strategy to replace traditional cooling devices such as air conditioners. However, most of these passive radiative coolers exhibit white or grey color to maximize the solar irradiance reflection. There is a gradual interest to develop colored daytime radiative cooling (CDRC)^53,119–121^ to provide an aesthetic appearance for radiative cooling that can better fit the environment. Guan *et al.*^78^ designed a transmissive type CDRC by stacking a selective emitter on top of a stepwise nanocavity. They first used the memetic algorithm to design the multilayer structure for a selective emitter, and then used tandem neural networks to design the nanocavity-based colored filter. However, this two-step design process may not give optimal performance.

To better balance the color appearance and cooling power, Keawmuang *et al.*^84^ proposed a new method to simultaneously consider the effect of emitters and the cavity materials on both color appearance and cooling capacity. Fig. 3(c) illustrates the schematic of designed colored radiative cooling made up of a selective emitter (SE) on top of a metal-insulator-metal (MIM) Fabry–Perot cavity. Instead of following the two-step design process, they focus on the simultaneous design of both reflection and emissivity spectra over the 300 nm to 2500 nm wavelength range: the reflection spectrum shows the desired color performance in the visible region while having close to 100% reflection in the infrared region. A tandem network was selected as their model backbone. Using this method, they successfully designed multiple CDRC with high cooling powers of 11.2, 38.3, and 24.1 W m^−2^ for yellow, magenta, and cyan colors, respectively. Fig. 3(d) shows the details of the reflection spectrum and cooling power of these three designed structures.

### Radio-frequency (RF)-transparent chrome-mimicking color

**Fig. 4:**
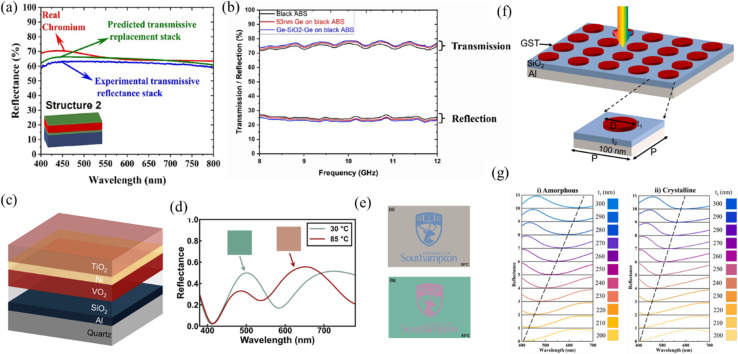
Examples of novel structural color applications. The reflection spectrum in the visible region (a) and their transmission spectrum in the radio-frequency region (b) for RF-transparent colorful devices. (c) The schematic of the five-layer thin film structure for a dynamic color device. (d) The reflective spectrum of the dynamic structure at 30 °C (cyan curve) and 85 °C (red curve). (e) The dynamic color change for the designed logo at 30 °C and 85 °C. (f) The schematic of the GST based dynamic color device. (g) The dynamic color change for the GST metamaterial when GST is in the (i) amorphous state and (ii) crystalline state, for different thicknesses of SiO_2_. (a and b) Reprinted from Saha *et al.*^49^ Copyright @ 2023, American Chemical Society. (c–e) Reprinted from Dai *et al.*^76^ Copyright @ 2022, Optical Publishing Group. (f and g) Reprinted from Prakash *et al.*^79^ Copyright @ 2023, Elsevier.

In addition to extending structural color functionality to near-infrared and infrared, recently, Saha *et al.*^49^ combined reinforcement learning with inverse design and successfully identified a multi-layer thin film structure that is functional in both the visible region and radio-frequency (RF) region. They seek to find some replacements for decorative chrome plating, which has an appealing metallic appearance and is ubiquitous in everyday finishing and coatings, while their industrial fabrication process can lead to adverse health effects and cause environmental damage. To find an ideal design, they utilized reinforcement learning to perform an automatic design process and identified multiple structures that resemble the appearance of chrome color. As shown in Fig. 4(a), the reflection spectrum of the designed structure is close to that of the real chrome, which can be used for toxic chrome plating replacement in daily decorations. In addition, they avoid using metallic materials during design, which makes the structure transparent in the RF region and suitable for electronic integration because it will not block RF signals. Fig. 4(b) shows the transmission and reflection spectra of the fabricated structures in the RF region. From this perspective, researchers could explore other possibilities of new colors with an aesthetic appearance for RF devices.

### Dynamic structural color devices

In addition to designing multi-functional structures by bringing colorful appearance to existing photonic devices, another active research direction is to design dynamic structural color devices,^2,8,122–128^ which can be applied in color display, steganography, information encryption, *etc.* One way to achieve dynamic color switching is using phase-changing materials. For example, Dai *et al.*^76^ proposed to use GAN to design a dynamic structural color based on the insulator-metal transition in vanadium dioxide. As shown in Fig. 4(c), the designed structure is a thin-film structure with an asymmetric F–P cavity inside, and consists of the TiO_2_/Ni/VO_2_/SiO_2_/Al stack. When changing the temperature from 30° to 85°, the phase change of VO_2_ leads to a change in the reflection spectrum, and ultimately the dynamic structural color (see Fig. 4(d and e)). In another study, Prakash *et al.*^79^ utilized the bidirectional tandem neural networks to design dynamically tunable structural colors using a reflective metamaterial absorber, Ge_2_Sb_2_Te_5_ (GST). Fig. 4(f) shows the metasurface structure that consists of periodical GST nanodisks on top of the SiO_2_ and Al substrate. The phase change of GST leads to a refractive index change, thus changing the reflection spectrum. More detailed spectrum and color visualization can be found in Fig. 4(g).

In addition to phase changing materials, dynamic structural colors can also be achieved by adjusting the polarization^75^ of incident light, varying lateral strains,^83^*etc.* For example, Hu *et al.*^75^ proposed a polarization-controlled structural color based on hybrid metal–dielectric metasurfaces. The nanostructure consists of four rectangular nanoarrays and their resonance wavelength will shift accordingly as the polarization of incident light changes. They used tandem neural networks to first predict the color of the mixed metasurface and then inverse design the structural parameters for the target color. In ref. 83, the authors proposed lateral hybrid metasurface structural color devices, which are composed of metal and dielectric resonators sitting inside a stretchable medium. By applying lateral forces, the gap between each resonator will increase, thus changing the resonance condition and modifying the reflective color. Similarly, a fully connected tandem network was used to finish this complicated design process.

As a summary, in this section, we provide several examples demonstrating how deep learning can be used to address novel tasks in structural color applications beyond traditional color filters. With the popular research trends transitioning from single-functional nanostructures into multifunctional metasurfaces and dynamic devices, deep learning-based inverse design has great potential to bring structural color properties to other significant application domains,^129,130^ such as displays, sensing, encryption, and reconfigurable optics. We believe that there are still numerous applications that have both scientific and practical significance but remain underexplored. We encourage researchers and engineers to investigate these opportunities with help from deep learning. By leveraging the superior power of learning and exploration, one could be surprised by how much we could achieve using deep learning.

### Beyond efficient design: new capabilities

The most obvious advantage of deep learning-based inverse design compared to optimization is the design speed: once learned from training datasets, these neural network models can finish each inverse design task within 1 millisecond, which is much faster than the iterative optimization process. However, preparing such large-scale training datasets requires thousands or millions of simulations beforehand, which is time-consuming. In addition, training deep learning models also requires extensive computation resources. Considering these extra efforts, there has been a criticism of whether deep learning-based inverse design is worthwhile. In this section, we would like to resolve this issue by exploring new capabilities that deep learning-based methods could potentially bring to the field of color inverse design, including design diversity, design robustness, explainability, *etc.* We also discuss other promising capabilities to provide ideas and insights for readers.

### Diversity

Design diversity means whether an inverse design method could give multiple structures that are diverse from each other. This relates to the one-to-many mapping issue: because different structures could exhibit similar visual color appearance, for a specific color design (either reflective or transmissive type), we should also expect multiple candidate structures in the design space. An inverse design method that can identify a diverse set of design solutions would definitely be more physical. Furthermore, some structures may be impractical to make when considering the fabrication techniques or lab conditions, *e.g.*, if the designed structure has a very thin layer of material, it might be difficult to control the precise thickness through deposition. Diverse designs can benefit the practical fabrication process by providing more candidate structures. This issue cannot be solved very well using optimization-based methods because most optimization algorithms can only identify a single design as the output. In order to obtain multiple designs, we need to rerun the same algorithm starting from different random seeds and finish the whole optimization process again, which can be time-consuming.

However, it is straightforward for learning-based methods to provide diverse designs, especially for some generative-type neural networks. Both VAE and GAN explicitly model the conditional distribution by sampling from a normalized latent space, while VAE learns to generate diverse outputs by first encoding inputs into a latent distribution and then decoding them back under the target condition, and GAN uses a generator to produce different structures based on sampled random vectors. On the other hand, sequence models, including RNN and transformers, handle this ambiguity through an autoregressive sampling process: given a design target, these models output a probability distribution over the design space; sampling from this distribution would generate different sets of structures with the desired performance. Unlike optimization-based methods, all these probabilistic learning frameworks inherently embed diversity into their generation mechanism, making them more physical and general.

**Fig. 5:**
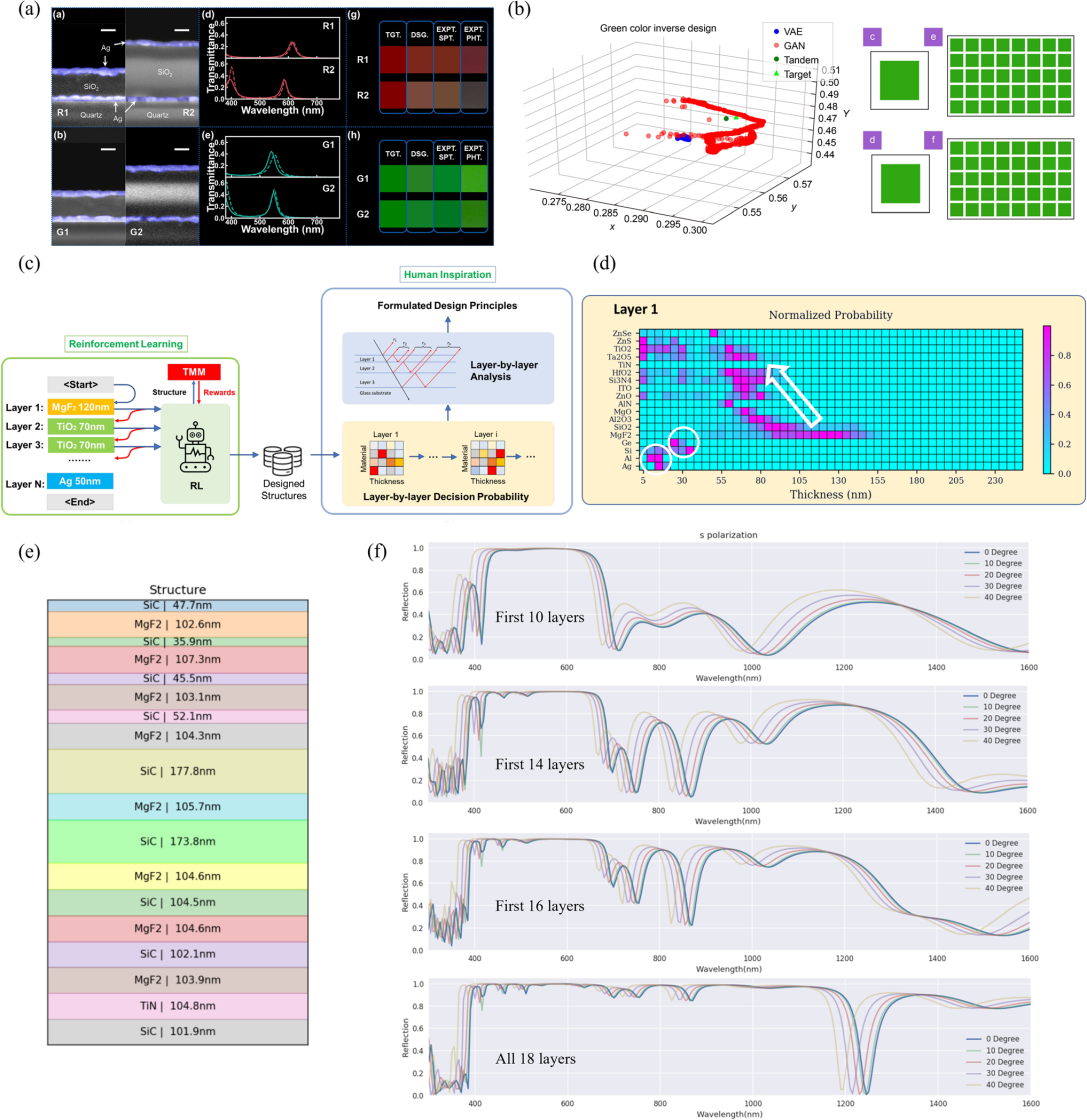
Examples of new capabilities from deep learning-based structural color. (a) Multiple structures designed by c-GAN with similar color performances. The structure is based on a three-layer FP cavity. Experimental results also verified the structural color appearance. (b) Benchmark results for design diversity of three different models: GAN, VAE and tandem network. (c) Schematic illustration of using reinforcement learning to help identify and explain the design principle for multilayer-based structural color. (d) Example of the probability of selecting the material and thickness for the first layer. We can find that optimal designs are clustered in three regions (white marks) based on the type of the materials: metal, dielectric, or semiconductor. (e) An ultra-broadband high reflector designed by an RL algorithm. The top medium is air, and the bottom medium is a glass substrate. (f) Layer-by-layer reflection analysis shows that the designed structure intrinsically forms multiple small reflectors within 18 layers and merges to become a ultra-broadband reflector. (a) Reprinted from Dai *et al.*^93^ Copyright @ 2022, Elsevier. (b) Reprinted from Ma *et al.*^94^ Copyright @ 2022, Institute of Optics and Electronics, Chinese Academy of Sciences. (c–f) Reprinted from Ma *et al.*^107^ Copyright @ 2024, University of Michigan.

For example, Dai *et al.*^93^ proposed to use conditional GAN to find multiple solutions for structural color. The nanostructure is based on a transmissive type Ag/SiO_2_/Ag FP-cavity. In conditional GAN (c-GAN), a latent vector that is sampled from a normal distribution is introduced during the design process, and each sampling process would lead to a different structure design. As shown in Fig. 5(a), using this method, the authors have identified several distinct structures for different colors. The experimental results also verified their design. In a benchmark study, Ma *et al.*^36^ compared the diversity of different neural networks for structural color inverse design, and also showed that conditional GANs give the best design diversity (see Fig. 5(b)). In addition, sequence generation networks, such as RNN and transformers, also naturally incorporate diversity during the generation process.^48^ For example, Saha *et al.*^49^ utilized RNN with a reinforcement learning algorithm to solve for chrome color inverse design and identified multiple thin film structures that behave the same. The reinforcement learning algorithm can dynamically explore inside the design space, which automatically incorporates structure diversity during the design process.

### Robustness

One of the major concerns for inverse design is the discrepancy between simulation and experimental results. There are multiple reasons, including fabrication errors, inaccurate material properties, measurement limitations, *etc.* A robust algorithm should be able to give some structures that are tolerable to these unexpected errors and minimize the performance discrepancy. In a benchmark study,^36^ the authors extensively compared the robustness of three neural networks (tandem network, VAE, and GAN) based on two aspects. First, they examined the robustness of neural network output by checking if the predicted structures are physical, *e.g.*, satisfy certain structural requirements. Second, they also examined the robustness of designed structures with respect to fabrication errors. To do so, they intentionally added some fabrication errors to the designed structures by slightly changing the structural parameters, then simulated their optical responses, and compared the performance drop. Their results have shown that VAE is the most robust model for inverse design. In addition to this work, we have not found much other related work in this important direction. We hope that there can be more breakthroughs in the future.

### Explainability

Apart from diversity and robustness, researchers are also interested to know why the inverse design algorithm prefers to give certain structures, instead of other structures, and try to understand the hidden physics behind these designed structures. Although many neural networks are black-box, there are already some studies trying to uncover the hidden mechanism. In ref. 107, the authors proposed a novel method to identify general design principles in color inverse design by leveraging the sequential generation process in reinforcement learning. The ideas are illustrated in Fig. 5(c). To do so, they first run the reinforcement learning algorithm for inverse design and collect multiple diverse sets of high-performance structures identified by the algorithm. Then, they combine these structures together and calculate the probability distribution when designing each specific layer, which leads to a layer-by-layer decision probability matrix (see Fig. 5(d)). Based on this, they can conduct a layer-by-layer physical analysis based on light propagation and interface interaction. With this method, they have successfully identified three different design principles for a reflective chrome color.

Furthermore, the authors used a similar algorithm to design an ultra-broadband high reflector and identified a novel structure. Although a Distributed Bragg Reflector (DBR)^131,132^ is widely used as a high reflector, its bandwidth is limited by the refractive index in the alternating layers. A general way to extend the bandwidth is to create a heterostructure DBR by vertically stacking multiple DBRs with different working wavelengths,^133^ which requires a significantly higher number of layers. Using RL algorithms, the authors designed a thin film structure with only 18 layers, which exhibits a high reflection extending from 400 nm to 1200 nm. The designed structure is shown in Fig. 5(e). By simulating the optical responses of the AI-generated structures, the physical mechanism was uncovered: by intrinsically forming multiple DBRs with different bandwidths and resonance wavelengths, with each resonance corresponding to different layers inside the structure, the high-reflection region starts to overlap with each other and form an ultra-broadband reflector. This structure suggests a novel design principle for an ultra-broadband high reflector.

To better understand the identified design principle, Fig. 5(f) shows the layer-by-layer reflection spectrum for the designed structure with the first 10, 14, 16, and 18 layers (counting from the top), respectively. We can see that for the first 10 layers, a 200 nm-width high reflection region at around 500 nm is already established, although the reflection in the near-infrared region is still limited. When adding more layers, these reflection peaks at 800 nm, 900 nm, and 1200 nm start to emerge and they start to line up together to form a wide reflection region. Finally, when adding all 18 layers, all these reflection peaks reach a maximum and line together to give an ultra-broad reflection region. This suggests the possibility that using deep learning methods, one can potentially identify novel design and structures that can solve existing difficulties.

In addition, there are already some techniques developed in deep learning society to explain the relationship between input and output to better understand predictions from neural networks. The SHAP value is one such method.^134,135^ We hope that in the future, more methods and ideas can be brought in to further improve the explainability of structural color inverse design.

## Summary

In conclusion, this perspective gives a high-level summary of research frontiers in deep learning-based inverse design for structural colors, and provides insights into topics to explore as future research directions. Traditional optimization-based methods are time-consuming and may fail to identify ideal nanostructures with good performance. In addition, it is difficult for these methods to incorporate new capabilities such as diversity, robustness, or explainability into design considerations. Differently, deep learning solves the problem by learning a general mapping from color space to structure space, and can tackle each design task efficiently. Although there are criticisms about resources used for the dataset preparation and model training, deep learning can bring plenty of new capabilities to this domain. We have identified multiple potential areas that worth exploring and would like to highlight the great potential here.

On the other hand, with the strong capabilities of deep learning, we would also like to encourage researchers to expand the scope of structural color applications. Moving beyond conventional color filters, we have identified some new opportunities around areas including colored solar cells, colored radiative cooling devices, multifunctional RF devices, and new dynamic structural colors. Most of these applications are related to providing aesthetic appearances to existing optical structures and devices, making them multi-functional and deployed more broadly. By embracing interdisciplinary interactions between deep learning and inverse design, we believe that the field of structural color inverse design will experience significant improvement and innovations.

## Author contributions

All authors contributed to the writing and revision of the manuscript and have approved the final version of the perspective.

## Conflicts of interest

There are no conflicts to declare.

## Data Availability

No primary research results, software or code have been included, and no new data were generated or analysed as part of this perspective.

## References

[cit1] Sun J., Bhushan B., Tong J. (2013). RSC Adv..

[cit2] Wang D., Liu Z., Wang H., Li M., Guo L. J., Zhang C. (2023). Nanophotonics.

[cit3] Kinoshita S., Yoshioka S., Miyazaki J. (2008). Rep. Prog. Phys..

[cit4] Xuan Z., Li J., Liu Q., Yi F., Wang S., Lu W. (2021). Innovation.

[cit5] Kinoshita S., Yoshioka S. (2005). ChemPhysChem.

[cit6] Feng W.-J., Paik J., Jay Guo L. (2024). Mater. Chem. Front..

[cit7] Xu T., Shi H., Wu Y.-K., Kaplan A. F., Ok J. G., Guo L. J. (2011). Small.

[cit8] Wang W., Wang L., Fu Q., Zhang W., Wang L., Liu G., Huang Y., Huang J., Zhang H., Guo F., Wu X. (2025). Opto-Electron. Sci..

[cit9] Zi J. , Yu X., Li Y., Hu X., Xu C., Wang X., Liu X. and Fu R., Coloration strategies in peacock feathers, 2003, vol. 100, no. 22, pp. 12576–12578 10.1073/pnas.2133313100 PMC240659 14557541

[cit10] Seago A. E., Brady P., Vigneron J.-P., Schultz T. D. (2008). J. R. Soc., Interface.

[cit11] Yang Z., Ji C., Liu D., Guo L. J. (2019). Adv. Opt. Mater..

[cit12] Ma T., Ma M., Guo L. J. (2025). iScience.

[cit13] Liu Y., Wang H., Ho J., Ng R. C., Ng R. J. H., Hall-Chen V. H., Koay E. H. H., Dong Z., Liu H., Qiu C.-W., Greer J. R., Yang J. K. W. (2019). Nat. Commun..

[cit14] Su X., Xia H., Zhang S., Tang B., Wu S. (2017). Nanoscale.

[cit15] Keshavarz Hedayati M., Elbahri M. (2017). Plasmonics.

[cit16] Chang S., Guo X., Ni X. (2018). Annu. Rev. Mater. Res..

[cit17] Song M., Wang D., Peana S., Choudhury S., Nyga P., Kudyshev Z. A., Yu H., Boltasseva A., Shalaev V. M., Kildishev A. V. (2019). Appl. Phys. Rev..

[cit18] Xue J., Zhou Z.-K., Wei Z., Su R., Lai J., Li J., Li C., Zhang T., Wang X.-H. (2015). Nat. Commun..

[cit19] Xu T., Wu Y.-K., Luo X., Guo L. J. (2010). Nat. Commun..

[cit20] Kristensen A., Yang J. K. W., Bozhevolnyi S. I., Link S., Nordlander P., Halas N. J., Mortensen N. A. (2016). Nat. Rev. Mater..

[cit21] Liu T., Liu T., Gao F., Glotzer S. C., Solomon M. J. (2022). J. Phys. Chem. B.

[cit22] Takeoka Y., Yoshioka S., Teshima M., Takano A., Harun-Ur-Rashid M., Seki T. (2013). Sci. Rep..

[cit23] Wang H., Liu Y., Chen Z., Sun L., Zhao Y. (2020). Sci. Adv..

[cit24] Tikhonravov A. V., Trubetskov M. K., DeBell G. W. (2007). Appl. Opt..

[cit25] Rabady R. I., Ababneh A. (2014). Optik.

[cit26] Schubert M. F., Mont F. W., Chhajed S., Poxson D. J., Kim J. K., Schubert E. F. (2008). Opt. Express.

[cit27] Ma W., Liu Z., Kudyshev Z. A., Boltasseva A., Cai W., Liu Y. (2021). Nat. Photonics.

[cit28] Liu Z., Zhu D., Raju L., Cai W. (2021). Adv. Sci..

[cit29] Jiang J., Chen M., Fan J. A. (2021). Nat. Rev. Mater..

[cit30] Wiecha P. R., Arbouet A., Girard C., Muskens O. L. (2021). Photonics Res..

[cit31] Hegde R. S. (2020). Nanoscale Adv..

[cit32] Khaireh-Walieh A. , Langevin D., Bennet P., Teytaud O., Moreau A. and Wiecha P. R. 10.1515/nanoph-2023-0527 PMC11501815 39634708

[cit33] Kang M., Choi S., Fu K., Liu X., Wei Z., Jin L., Wang H., Martin O. J. F., Yang J. K. W., So S., Badloe T. (2026). Opto-Electron. Sci..

[cit34] Inverse Design in Nanophotonics via Representation Learning - Marzban - 2026 - Advanced Optical Materials, Wiley Online Library, https://advanced.onlinelibrary.wiley.com/doi/10.1002/adom.202502062, accessed June 5, 2026

[cit35] Ueno A., Hu J., An S. (2024). npj Nanophoton..

[cit36] Taigao M., Mustafa T., Haozhu W., Jay G. L. (2022). Opto-Electron. Sci..

[cit37] Kim J. , Li M., Hinder O. and Leu P., 37th Conference on Neural Information Processing Systems (NeurIPS 2023) Track on Datasets and Benchmarks, https://proceedings.neurips.cc/paper_files/paper/2023/file/0f12c9975ff4f2e44a5a26ef01b0b249-Paper-Datasets_and_Benchmarks.pdf

[cit38] Schneider P.-I., Garcia Santiago X., Soltwisch V., Hammerschmidt M., Burger S., Rockstuhl C. (2019). ACS Photonics.

[cit39] Ren S., Mahendra A., Khatib O., Deng Y., Padilla W. J., Malof J. M. (2022). Nanoscale.

[cit40] S. K. Shevell , in The Science of Color, Second Edition, Elsevier Science Ltd, Amsterdam, 2003

[cit41] Conway B. R., Chatterjee S., Field G. D., Horwitz G. D., Johnson E. N., Koida K., Mancuso K. (2010). J. Neurosci..

[cit42] Wilms L., Oberfeld D. (2018). Psychol. Res..

[cit43] Priest I. G. (1924). J. Opt. Soc. Am..

[cit44] Camgöz N., Yener C., Güvenç D. (2002). Color Res. Appl..

[cit45] Fay C. D., Wu L. (2024). Talanta.

[cit46] Ohno Y. (2000). NIP & Digital Fabrication Conference.

[cit47] Smith T., Guild J. (1931). Trans. Opt. Soc..

[cit48] Ma T., Wang H., Guo L. J. (2024). Opto-Electron. Adv..

[cit49] Saha A., Ma T., Wang H., Guo L. J. (2023). ACS Appl. Mater. Interfaces.

[cit50] Li M. , Kim J., Hinder O. and Leu P., CHROMA: Conversational Human-Readable Optical Multilayer Assembly for Natural Language-Driven Inverse Design of Structural Coloration, in AI for Accelerated Materials Design-NeurIPS 2025, 2025, https://neurips.cc/virtual/2025/loc/san-diego/128969

[cit51] Schanda J. , Colorimetry: Understanding the CIE System, John Wiley & Sons, 2007

[cit52] Li Z., Li S., Yan J., Peng J., Ma T. (2025). Nat. Rev. Clean Technol..

[cit53] Xie B., Liu Y., Xi W., Hu R. (2023). Mater. Today Energy.

[cit54] Jablonka K. M., Schwaller P., Ortega-Guerrero A., Smit B. (2024). Nat. Mach. Intell..

[cit55] Boiko D. A., MacKnight R., Kline B., Gomes G. (2023). Nature.

[cit56] A review of large language models and autonomous agents in chemistry - Chemical Science, RSC Publishing, 10.1039/D4SC03921A, https://pubs.rsc.org/en/content/articlehtml/2025/sc/d4sc03921a, accessed December 30, 2025 PMC11739813 39829984

[cit57] Hu M. , Ma C., Li W., Xu W., Wu J., Hu J., Li T., Zhuang G., Liu J., Lu Y., Chen Y., Zhang C., Tan C., Ying J., Wu G., Gao S., Chen P., Lin J., Wu H., Chen L., Wang F., Zhang Y., Zhao X., Tang F., Su E., Ning J., Liu X., Du Y., Ji C., Jiang P., Tang C., Huang Z., Liu J., Wei J., Yang Y., Zhang X., Wang G., Yang Y., Xu H., Chen Z., Wang Y., Tang C., Wu J., Ren Y., Yan S., Wang Z., Xu Z., Su S., Sun S., Zhao R., Zhang Z., Yang D., Wei J., Wang J., Xu J., Yan J., Tang W., Zhu H., Liu Y., Wang F., Shen Y., Ji Y., Su Y., Xie T., Shan H., Feng C.-M., Hou Z., Song D., Liu L., Huang Y., Yu L., Fu B., Wang S., Li X., Hu X., Gu Y., Fei B., Wang B., Cao Y., Shen M., Xu J., Duan H., Yan F., Hao H., Li J., Du J., Wang Y., Razzak I., Deng Z., Zhang C., Wu L., He C., Lu Z., Huang J., Shao W., Liu Y., Luo S., Xin Y., Liu X., Ling F., Li Y., Wang A., Sun S., Zheng Q., Dong N., Fu T., Zhou D., Lu Y., Zhang W., Ye J., Cai J., Chen Y., Ouyang W., Qiao Y., Ge Z., Tang S., He J., Song C., Bai L. and Zhou B., arXiv, 2025, preprint, arXiv:2508.21148, 10.48550/arXiv.2508.21148

[cit58] Gao S., Fang A., Huang Y., Giunchiglia V., Noori A., Schwarz J. R., Ektefaie Y., Kondic J., Zitnik M. (2024). Cell.

[cit59] Gridach M. , Nanavati J., Abidine K. Z. E., Mendes L. and Mack C., arXiv, 2025, preprint, arXiv:2503.08979, DOI: 10.48550/arXiv.2503.08979

[cit60] Yuan W., Chen G., Wang Z., You F. (2025). Adv. Mater..

[cit61] Zhang L., Liu Z., Ni B., Wang Q. (2026). Adv. Funct. Mater..

[cit62] Liu F. , Yao Y., Guo P., Yang Z., Zhao Z., Lin X., Tong X., Yuan M., Lu Z., Wang Z. and Zhang Q., arXiv, 2024, preprint, arXiv:2410.14716, DOI: 10.48550/arXiv.2410.14716

[cit63] Kim M. , Park H. and Shin J.

[cit64] Mandal I., Soni J., Zaki M., Smedskjaer M. M., Wondraczek K., Wondraczek L., Gosvami N. N., Krishnan N. M. A. (2025). Nat. Commun..

[cit65] Gao L., Li X., Liu D., Wang L., Yu Z. (2019). Adv. Mater..

[cit66] Huang Z., Liu X., Zang J. (2019). Nanoscale.

[cit67] Sajedian I., Badloe T., Rho J. (2019). Opt. Express.

[cit68] Dai P., Wang Y., Hu Y., de Groot C. H., Muskens O., Duan H., Huang R. (2021). Photon. Res..

[cit69] Roberts N. B., Keshavarz Hedayati M. (2021). Appl. Phys. Lett..

[cit70] Ma L., Hu K., Wang C., Yang J.-Y., Liu L. (2021). Nanomaterials.

[cit71] Zhou Y., Hu L., Wang C., Ma L. (2022). Nanomaterials.

[cit72] Wang J., Lin Z., Fan Y., Mei L., Deng W., Lv J., Xu Z. (2022). Materials.

[cit73] Wang H., Guo L. J. (2022). iScience.

[cit74] Hao Y., Liu Y., Wu T., Li J., Sun Y., Wang Y., Fan H., Wang X., Ye H. (2022). Opt. Commun..

[cit75] Hu L., Ma L., Wang C., Liu L. (2022). Opt. Express.

[cit76] Dai P., Sun K., Muskens O. L., de Groot C. H., Huang R. (2022). Opt. Mater. Express.

[cit77] Liu C., Zhang J., Zhao Y., Ai B. (2023). Adv. Intell. Syst..

[cit78] Guan Q., Raza A., Mao S. S., Vega L. F., Zhang T. (2023). ACS Photonics.

[cit79] P. S R., Kumar R., Mitra A. (2023). Photon. Nanostruct: Fundam. Appl..

[cit80] Clini de Souza A., Lanteri S., Hernández-Figueroa H. E., Abbarchi M., Grosso D., Kerzabi B., Elsawy M. (2023). Sci. Rep..

[cit81] Hu Y., Zhang W., Chen Y., Zuo H., Tian M., Tang M., Li L., Xie Z., Huang Y. (2024). Results Phys..

[cit82] Yan H., Hao R., Meng Y., Jin S. (2024). Appl. Opt..

[cit83] Fang R., Ghasemi A., Zeze D., Hedayati M. K. (2024). RSC Adv..

[cit84] Keawmuang H., Badloe T., Lee C., Park J., Rho J. (2024). Sol. Energy Mater. Sol. Cells.

[cit85] Zuo Y., Ni B., Zhou Y., Guo J., Ni H., Zhou X., Haque S. J., Chang J. (2024). J. Opt. Soc. Am. B.

[cit86] Wang Z.-D., Meng Y.-L., Li Y., Gao H., Zhang T., Pan G.-M., Kang J., Zhan C.-L. (2024). Opt. Commun..

[cit87] Yang H., Ni B., Guo J., Zhou H., Chang J. (2025). Chin. Phys. B.

[cit88] Sadman S., Khaleque A., Islam R., Sayed R. (2025). Opt. Mater. Express.

[cit89] Lu K., Chen L., Li C., Zhu H., Wang C., Ma L. (2025). Photon. Nanostruct: Fundam. Appl..

[cit90] Gao L., Li X., Liu D., Wang L., Yu Z. (2019). Adv. Mater..

[cit91] Ma L., Hu K., Wang C., Yang J.-Y., Liu L. (2021). Nanomaterials.

[cit92] Zhou Y., Hu L., Wang C., Ma L. (2022). Nanomaterials.

[cit93] Dai P., Sun K., Yan X., Muskens O. L., (Kees) de Groot C. H., Zhu X., Hu Y., Duan H., Huang R. (2022). Nanophotonics.

[cit94] Ma T. , Tobah M., Wang H., Guo L. J., Department of Physics, The University of Michigan, Ann Arbor, Michigan 48109, USA, Department of Materials Science and Engineering, The University of Michigan, Ann Arbor, Michigan 48109, USA, and Department of Electrical Engineering and Computer Science, The University of Michigan, Ann Arbor, Michigan 48109, USA, OES, 2022, 1, 210012

[cit95] Wang H., Zheng Z., Ji C., Jay Guo L. (2021). Mach. Learn.: Sci. Technol..

[cit96] Dey R. and Salem F. M., in 2017 IEEE 60th International Midwest Symposium on Circuits and Systems (MWSCAS), 2017, pp. 1597–1600

[cit97] Chung J. , Gulcehre C., Cho K. and Bengio Y., arXiv, 2014, preprint, arXiv:1412.3555, DOI: 10.48550/arXiv.1412.3555

[cit98] Vaswani A., Shazeer N., Parmar N., Uszkoreit J., Jones L., Gomez A. N., Kaiser Ł., Polosukhin I. (2017). Adv. Neural Inf. Process. Syst..

[cit99] Ouyang L., Wu J., Jiang X., Almeida D., Wainwright C., Mishkin P., Zhang C., Agarwal S., Slama K., Ray A., Schulman J., Hilton J., Kelton F., Miller L., Simens M., Askell A., Welinder P., Christiano P. F., Leike J., Lowe R. (2022). Adv. Neural Inf. Process. Syst..

[cit100] DeepSeek-AI , Guo D., Yang D., Zhang H., Song J., Zhang R., Xu R., Zhu Q., Ma S., Wang P., Bi X., Zhang X., Yu X., Wu Y., Wu Z. F., Gou Z., Shao Z., Li Z., Gao Z., Liu A., Xue B., Wang B., Wu B., Feng B., Lu C., Zhao C., Deng C., Zhang C., Ruan C., Dai D., Chen D., Ji D., Li E., Lin F., Dai F., Luo F., Hao G., Chen G., Li G., Zhang H., Bao H., Xu H., Wang H., Ding H., Xin H., Gao H., Qu H., Li H., Guo J., Li J., Wang J., Chen J., Yuan J., Qiu J., Li J., Cai J. L., Ni J., Liang J., Chen J., Dong K., Hu K., Gao K., Guan K., Huang K., Yu K., Wang L., Zhang L., Zhao L., Wang L., Zhang L., Xu L., Xia L., Zhang M., Zhang M., Tang M., Li M., Wang M., Li M., Tian N., Huang P., Zhang P., Wang Q., Chen Q., Du Q., Ge R., Zhang R., Pan R., Wang R., Chen R. J., Jin R. L., Chen R., Lu S., Zhou S., Chen S., Ye S., Wang S., Yu S., Zhou S., Pan S., Li S. S., Zhou S., Wu S., Ye S., Yun T., Pei T., Sun T., Wang T., Zeng W., Zhao W., Liu W., Liang W., Gao W., Yu W., Zhang W., Xiao W. L., An W., Liu X., Wang X., Chen X., Nie X., Cheng X., Liu X., Xie X., Liu X., Yang X., Li X., Su X., Lin X., Li X. Q., Jin X., Shen X., Chen X., Sun X., Wang X., Song X., Zhou X., Wang X., Shan X., Li Y. K., Wang Y. Q., Wei Y. X., Zhang Y., Xu Y., Li Y., Zhao Y., Sun Y., Wang Y., Yu Y., Zhang Y., Shi Y., Xiong Y., He Y., Piao Y., Wang Y., Tan Y., Ma Y., Liu Y., Guo Y., Ou Y., Wang Y., Gong Y., Zou Y., He Y., Xiong Y., Luo Y., You Y., Liu Y., Zhou Y., Zhu Y. X., Xu Y., Huang Y., Li Y., Zheng Y., Zhu Y., Ma Y., Tang Y., Zha Y., Yan Y., Ren Z. Z., Ren Z., Sha Z., Fu Z., Xu Z., Xie Z., Zhang Z., Hao Z., Ma Z., Yan Z., Wu Z., Gu Z., Zhu Z., Liu Z., Li Z., Xie Z., Song Z., Pan Z., Huang Z., Xu Z., Zhang Z. and Zhang Z., DeepSeek-R1, https://arxiv.org/abs/2501.12948v1, accessed May 23, 2025

[cit101] Touvron H. , Lavril T., Izacard G., Martinet X., Lachaux M.-A., Lacroix T., Rozière B., Goyal N., Hambro E., Azhar F., Rodriguez A., Joulin A., Grave E. and Lample G., arXiv, 2023, preprint, arXiv:2302.13971, DOI: 10.48550/arXiv.2302.13971

[cit102] Croitoru F.-A., Hondru V., Ionescu R. T., Shah M. (2023). IEEE Trans. Pattern Anal. Mach. Intell..

[cit103] Zhu L., Hua W., Lv C., Liu Y. (2025). Opt. Express.

[cit104] Seo D., Um S., Lee S., Ye J. C., Chung H. (2026). ACS Photonics.

[cit105] Mondal S., Park T., Biswas S., Wang A. X., Cai W. (2026). Nano Lett..

[cit106] Li J. , Guo J., Li Y., Mao Z., Shen J., Xu T., Das D., He J., Hu R., Lee Y., Tsuda K. and Shiomi J., CR-PHYS-SC, 10.1016/j.xcrp.2026.103174

[cit107] Ma T. , Thesis, University of Michigan, Ann Arbor, 2024

[cit108] Sullivan D. M. , Electromagnetic simulation using the FDTD method, John Wiley & Sons, 2013

[cit109] Hugonin J. P. and Lalanne P., arXiv, 2023, preprint, arXiv:2101.00901, DOI: 10.48550/arXiv.2101.00901

[cit110] Byrnes S. J. , arXiv, preprin, arXiv:1603.02720, 10.48550/arXiv.1603.02720

[cit111] Deng L. and Yu D., Foundations and Trends in Signal Processing, 10.1561/2000000039

[cit112] LeCun Y., Bengio Y., Hinton G. (2015). Nature.

[cit113] Deng L. , A tutorial survey of architectures, algorithms, and applications for deep learning, APSIPA transactions on Signal and Information Processing, 2014, vol. 3, p. e2

[cit114] Pattern Recognition and Machine Learning

[cit115] Alpaydin E. , Introduction to Machine Learning, fourth edition, MIT Press, 2020

[cit116] Bing J., Caro L. G., Talathi H. P., Chang N. L., Mckenzie D. R., Ho-Baillie A. W. Y. (2022). Joule.

[cit117] Ballif C., Perret-Aebi L.-E., Lufkin S., Rey E. (2018). Nat. Energy.

[cit118] Zhang W., Anaya M., Lozano G., Calvo M. E., Johnston M. B., Míguez H., Snaith H. J. (2015). Nano Lett..

[cit119] Sheng C., An Y., Du J., Li X. (2019). ACS Photonics.

[cit120] Yoon T. Y., Son S., Min S., Chae D., Woo H. Y., Chae J.-Y., Lim H., Shin J., Paik T., Lee H. (2021). Mater. Today Phys..

[cit121] Son S., Jeon S., Chae D., Lee S. Y., Liu Y., Lim H., Oh S. J., Lee H. (2021). Nano Energy.

[cit122] Li X., Zhao J., Yang J., Huo Y., Yu Y. (2025). Adv. Sci..

[cit123] Ahmed A., Zhou S., Yu B., Li T., Bodin J. N., Zeng S., Jiang X., Sun L. (2025). Chem. Rev..

[cit124] Xuan Z., Li J., Liu Q., Yi F., Wang S., Lu W. (2021). Innovation.

[cit125] Kim Y., Lee J., Kim W.-S., Heo H., Jeon D., Yang B., Li X., Keawmuang H., Hu S., Kim Y.-K., Badloe T., Rho J. (2026). Opto-Electron. Sci..

[cit126] Tang P., Jeon Y., Li X., Kim Y., Seong J., Hu S., Kim K., Li G., Badloe T., Rho J. (2025). Adv. Opt. Mater..

[cit127] Wang H., Wang H., Ruan Q., Chan J. Y. E., Zhang W., Liu H., Rezaei S. D., Trisno J., Qiu C.-W., Gu M., Yang J. K. W. (2023). Nat. Nanotechnol..

[cit128] Li X., Cai X., Liu C., Kim Y., Badloe T., Liu H., Rho J., Xiao S. (2024). Opto-Electron. Sci..

[cit129] Wei Q., Sain B., Wang Y., Reineke B., Li X., Huang L., Zentgraf T. (2019). Nano Lett..

[cit130] Lim K. T. P., Liu H., Liu Y., Yang J. K. W. (2019). Nat. Commun..

[cit131] Wang S. (1974). IEEE J. Quantum Electron..

[cit132] Thompson R. C. , Optical waves in layered media, Taylor & Francis, 1990

[cit133] Wang X., Hu X., Li Y., Jia W., Xu C., Liu X., Zi J. (2002). Appl. Phys. Lett..

[cit134] Yeung C., Tsai J.-M., King B., Kawagoe Y., Ho D., Knight M. W., Raman A. P. (2020). ACS Photonics.

[cit135] Yeung C., Ho D., Pham B., Fountaine K. T., Zhang Z., Levy K., Raman A. P. (2022). ACS Photonics.

